# Influence of Thermal Parameters Related to Destabilization Treatments on Erosive Wear Resistance and Microstructural Variation of White Cast Iron Containing 18% Cr. Application of Design of Experiments and Rietveld Structural Analysis

**DOI:** 10.3390/ma12193252

**Published:** 2019-10-05

**Authors:** Alejandro Gonzalez-Pociño, Florentino Alvarez-Antolin, Juan Asensio-Lozano

**Affiliations:** Materials Pro Group, Departamento de Ciencia de los Materiales e Ingeniería Metalúrgica, Universidad de Oviedo, Independencia 13, 33004 Oviedo, Spain; UO204622@uniovi.es (A.G.-P.); jasensio@uniovi.es (J.A.-L.)

**Keywords:** high chromium white cast iron, erosive wear, secondary carbides, retained austenite, destabilization of austenite

## Abstract

High-Cr hypo-eutectic white cast irons are used in very demanding environments that require high resistance to erosive wear. The influence on the microstructural variation and erosive wear resistance of several fundamental factors related to the thermal treatments of these cast irons was analysed by means of a fractional Design of Experiments (DoE). These factors included the ones related to the destabilization of austenite. The precipitated phases were identified by X-ray diffraction (XRD), while the Rietveld structural refinement method was used to determine their percentages by weight. Erosion wear resistance was calculated using the test defined by ASTM G76. It was concluded that the quench cooling medium does not significantly influence either erosive wear resistance or the proportion of martensite or retained austenite. The destabilization temperature is a key factor with respect to the percentage of retained austenite. In order to increase the amount of martensite and decrease the amount of retained austenite, temperatures not exceeding 1000 °C are required. An increase of 100 °C in the destabilization temperature can lead to a 25% increase in retained austenite. Moreover, tempering temperatures of around 500 °C favour an additional increase in the percentage of martensite. Erosive wear commences on the matrix constituent without initially affecting the eutectic carbides. Once the deterioration of the matrix constituent surrounding these carbides occurs, they are released. High tempering times provide an increase in resistance to erosive wear due to a second destabilization of austenite during the said tempering.

## 1. Introduction

High-Cr hypoeutectic white cast irons are used in harsh environments that require high resistance to erosive wear [1]. Examples include the mining, cement and thermal power industries [2,3]. These cast irons show two microstructural peculiarities that condition their properties. The first is that the matrix phase of their eutectic constituent is made up of austenite, while the second is that the carbides which form part of the said eutectic are of the (Fe,Cr)_7_C_3_ type, also known as K_2_ carbides. These carbides show hardness values ranging between 1500 and 1800 HV [1,4,5]. Austenite has high hardenability, allowing its partial transformation into martensite via air cooling [5]. Furthermore, the austenite is found in the supersaturated state as a result of non-equilibrium solidification [6,7]. To enhance the wear resistance of these cast irons, it is advisable to carry out a treatment to destabilize this austenite, which entails an austenization treatment [8,9]. During this treatment, the precipitation of carbides is produced from the alloy elements rejected by the austenite, mainly chromium carbides of the K_2_ type [6,10,11]. As a result of this treatment, the increase in wear resistance and the reduction in retained austenite are favoured by the increase in the Ms (martensitic transformation temperature) [12,13]. If the destabilization temperature is low, e.g., 900 °C, the solubility limit of the C in the austenite decreases and hence the amount of secondary carbides increases [14]. The destabilization of austenite requires long dwell times at the austenization temperature. This is because of the high concentration of alloy elements in the austenite crystalline cell, which hinders the diffusion of carbon. As the dwell time at the destabilization temperature increases, two kinetics simultaneously compete with one another: on the one hand, increase in the density of secondary carbides; and on the other, dissolution of those eutectic carbides formed as a result of non-equilibrium solidification [15]. An excessive dwell time can lead to ‘‘thickening’’ of the secondary carbides [12]. Besides the austenite destabilization treatment, subsequent quenching and tempering treatment are required for the proper use of these cast irons. The tempering temperatures for martensite usually fall within the 200–250 °C range. However, it would be possible to achieve a second destabilization of the retained austenite at temperatures between 400 and 600 °C [4,16,17]. This would favour its transformation into martensite during cooling after this tempering [18]. Thus, the hardness and erosive wear resistance of these cast irons can be substantially enhanced by means of a suitable heat treatment [19]. The aim of this study was to analyse the hardness and erosive wear resistance of a hypo-eutectic white cast iron containing 18% Cr and 2% Mo by varying the process variables related to the thermal treatments of the said cast iron. A further aim was to correlate the results with the microstructural variation that the material undergoes. Specifically, parameters related to the destabilization of austenite were analysed, namely the temperature and dwell time at this destabilization temperature. Parameters related to the quench medium and tempering conditions were also analysed, such as temperature, tempering times and number of tempers. Table 1 shows the chemical composition of the white cast iron. The applied research method was a fractional Design of Experiments (DoE), analysing six factors and conducting eight experiments [20]. The results will allow manufacturers of this grade of cast iron to design the most suitable industrial heat treatment for this material to offer high hardness and high wear resistance.

## 2. Materials and Methods

The purpose of applying a Design of Experiments (DoE) was to deliberately modify certain working design parameters related to heat treatments, the aim being to generate changes in certain responses of the material. Specifically, the goal was to analyse the variations in hardness and erosive wear resistance as well as the resulting microstructural changes, subsequently correlating the results. The analysis of these changes allowed us to determine which of the working parameters have a significant effect on these responses. Table 2 shows the analysed parameters and the levels chosen to modify these working conditions in an orderly way. The DoE allows the effect of the variation of a factor on a given response to be determined. An example would be the effect on hardness of varying the destabilization of austenite temperature from 1000 to 1100 °C. The effect of the variation of a single factor is called a principal effect. Although the calculation of the effects is complex and laborious, it can be simplified using the Yates algorithm [20]. This algorithm can be straightforwardly implemented on a spreadsheet. The effect of one factor may often depend on the value that another takes; when this occurs, these factors are said to interact. The “weight” of the main effects on the variations is greater than that of the interactions of 2 factors, while the importance of the latter is in turn greater than that of the interactions of 3 factors, and so on. In industrial practice, it is sufficient to consider only the main effects and the 2-factor interactions, which enables the number of experiments to be reduced [20]. Based on this premise, 8 experiments were accordingly carried out in the present study, which supposes a 1/8 (64/8 = 8) fractional factorial design. If we wished to analyse all the possible interactions, we would need to perform 64 experiments (2^6^ = 64). In the case in hand, we estimated only 8 effects (2^6 − 3^). Table 3 shows the array of experiments thus generated to carry out a DoE with 6 factors, 2 levels and 8 experiments. Columns D, E and F have been respectively constructed from the product of columns A × B, A × C and B × C. The “Restricted Confounding Pattern” column indicates only the main effects and those 2-factor interactions whose effects are confounded with the main effects. The effects are linear combinations of the analysed responses. Hence, applying the central limit theorem (CLT), they will follow a normal law. If all the effects were non-significant, they would follow an N (0,σ) law and would thus appear aligned when represented on a normal probability plot. The normal probability plot scale makes it possible to convert the distribution function of the N (0,σ) law into a straight line. The coordinate point (0.50) is thus situated on this line. If any effect is significant, however, it will follow an N (μ,σ) law, not appearing aligned with the non-significant effects. Those effects that deviate from the straight line towards the ends on the normal probability plot are considered significant. For example, if an effect deviates to the left, this would indicate that the factor associated with this effect at its –1 level would increase the value of the response. Similarly, if an effect deviates to the right of the straight line, this would indicate that the factor associated with this effect at its +1 level would increase the value of the response [20]. The statistical analysis was carried out with the help of the Statgraphics Centurion XVI program, version 16.1.18.

The analysed responses were:

- The Vickers hardness. The applied load was of 981 N, while the hardness value was the average value obtained from 10 indentations.
- Erosive wear resistance. This test was carried out as per ASTM G76 [21] by means of compressed air blasting with corundum particles, applying a pressure of 4 bar, a flow rate of 120 g/min and a 30° angle of incidence on the sample surface. Three repetitions were performed per test. The duration of each test was 1 min. The abrasive particles were 50 microns in size and had an angular surface.
- The following microstructural variables:
  ▪ Percentage by weight of austenite
  ▪ Percentage by weight of martensite
  ▪ Percentage by weight of carbides
  ▪ Volume of the austenite crystal cell

## 3. Results and Discussion

**Figure 1:**
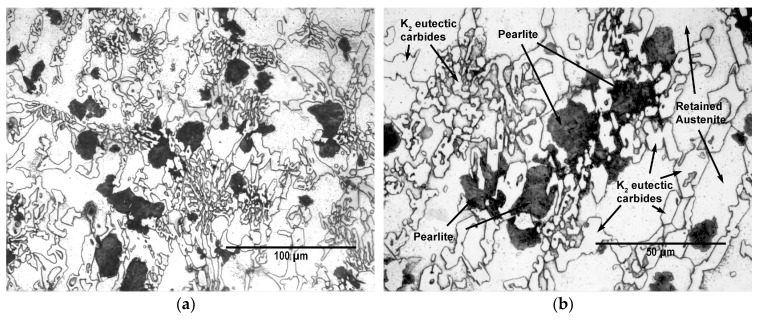
As-cast Microstructure: (**a**) ×500 magnification; (**b**) ×1000 magnification.

Figure 1 shows the microstructure of these cast irons in the as-cast state. This microstructure is mainly made up of eutectic carbides of the K_2_ type, retained austenite and pearlite.

**Figure 2:**
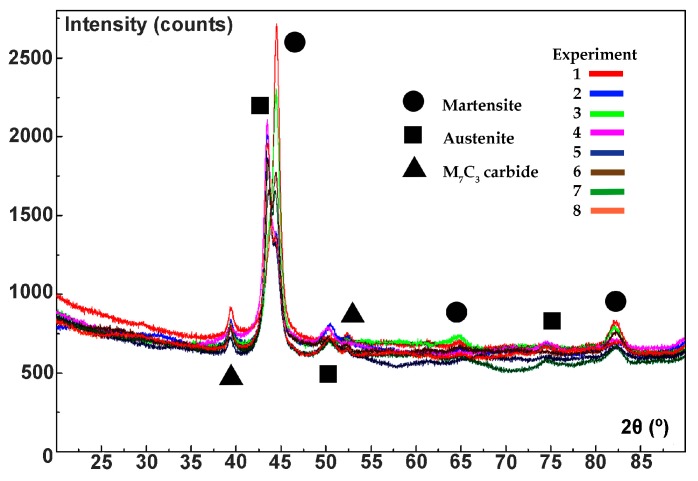
The diffractograms of the 8 experiments are shown in conjunction. The differences in intensities of the Bragg peaks associated with each of the experiments can be appreciated.

Figure 2 shows the diffractograms obtained in the 8 experiments. The analysis was carried out after having performed all the heat treatments indicated in Table 3 (array of experiments). The Bragg peaks corresponding to martensite were indexed to their reflections with Miller indices (110), (200) and (211). The Bragg peaks corresponding to austenite were indexed to their reflections with Miller indices (111), (200) and (222). Furthermore, other Bragg peaks can be appreciated on the irregular background produced by the fluorescence of the compositions that were identified with the structure of mixed carbides of type K_2_ (M_7_C_3_). The individual profile of each Bragg peak was fitted using pseudo-Voigt functions. Table 4 provides the 2θ and intensity (I) values of the Bragg peaks that stood out the most.

**Figure 3:**
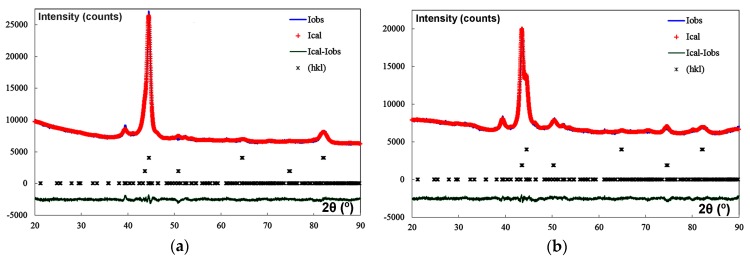

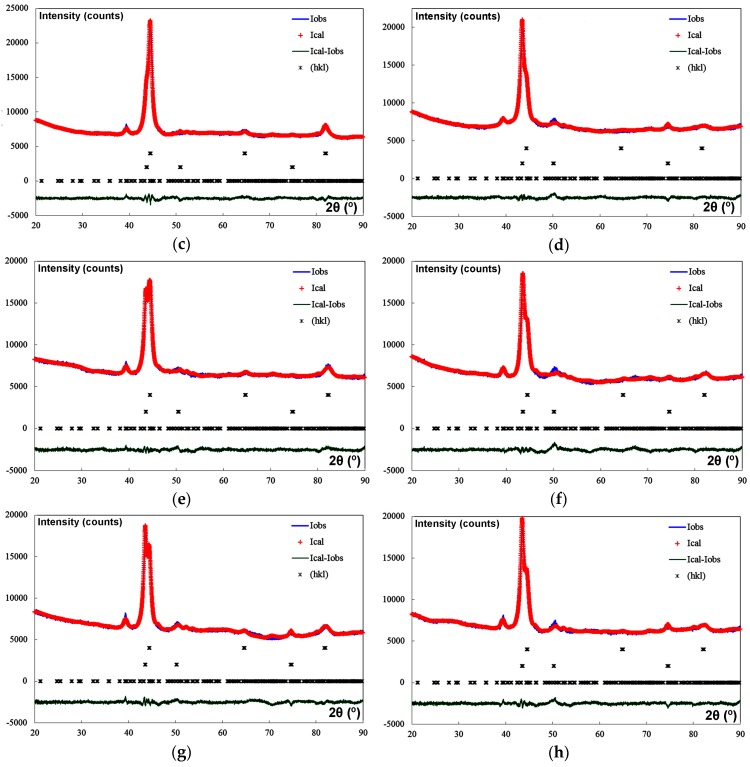
Overall fittings obtained by means of Rietveld structural refinement. The blue plots show the observed intensities and the red line, the intensities obtained using the Rietveld structural model. The dark green line indicates the difference between these intensities: (**a**) Experiment 1 (No.1); (**b**) Experiment 2 (No.2); (**c**) Experiment 3 (No.3); (**d**) Experiment 4 (No.4); (**e**) Experiment 5 (No.5); (**f**) Experiment 6 (No.6); (**g**) Experiment 7 (No.7); (**h**) Experiment 8 (No.8).

Figure 3 shows the overall fittings using the Rietveld method. Red crosses mark the observed intensities; the blue line, the intensity calculated according to the Rietveld structural model; the green line, the difference between the two; and the asterisks, the positions of the reflections.

Table 5 shows the percentages by weight and the network parameters of the main crystalline phases detected by XRD in each of the 8 experiments. The degree of accuracy of the fittings can be assessed by comparing the R_wp_ agreement factor and the R_exp_ index. The relationship between their squares, Chi^2^ = (R_wp_/R_exp_)^2^, is known as the goodness of fit. In our case, a large part of the obtained fittings reaches values around 2, which corroborates a high degree of certainty in the analysis.

**Figure 4:**
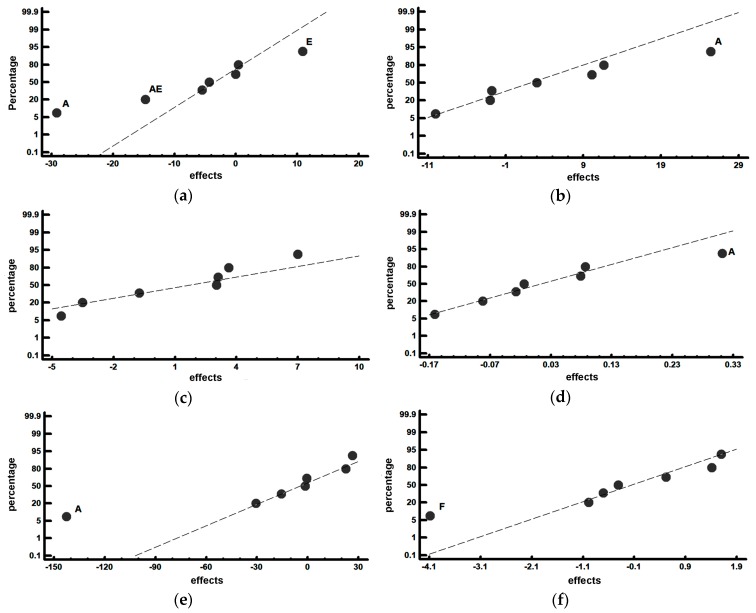
Representation of the effects on a normal probability plot. Those factors with a significant effect on the analysed responses are highlighted. (**a**) Percentage by weight of martensite; (**b**) Percentage by weight of austenite; (**c**) Percentage by weight of type K2 carbides; (**d**) Volume of the austenite crystal cell (Å^3^); (**e**) Vickers hardness; and (**f**) Weight loss in the erosive wear test (mg).

Table 6 shows the average values obtained in each experiment, together with the effects corresponding to the restricted confounding pattern specified in the array of experiments. The row corresponding to the average shows the average value obtained for each of the responses. Figure 4 shows the representation of these effects on a normal probability plot, highlighting those that have a significant effect on these responses.

Figure 4a shows that the main factors that have a significant effect on the percentage of martensite are Factors A (destabilization of austenite temperature) and E (tempering temperature). Thus, if the aim is to increase this percentage, both factors should be placed at their respective −1 and +1 levels; i.e., a destabilization temperature of 1000 °C and a tempering temperature of 500 °C. It appears to be confirmed that the austenite will be partially converted to martensite during cooling after tempering at 500 °C [4]. Figure 4a also shows the significant effect of the interaction of both factors, an increase in the percentage in martensite being produced when both factors are simultaneously placed at their respective −1 and +1 levels. Furthermore, Figure 4b shows that the destabilization temperature also has a significant effect on the percentage of retained austenite: placing this factor at 1100 °C leads to an increase in the percentage of austenite. Note that an increase of 100 °C in the destabilization temperature can lead to an increase of more than 25% in retained austenite. This value is similar to the increase in martensite (29%) that reducing these 100 °C induces.

**Figure 5:**
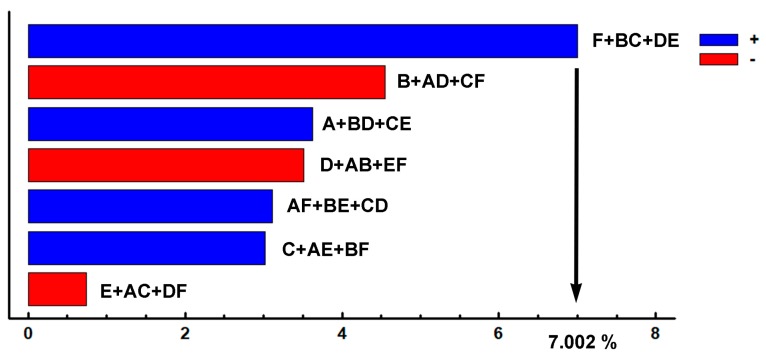
Pareto chart of the effects of each factor on the percentage by weight of K_2_ carbides. Placing Factor F at its +1 level (6-h tempering time) could produce a 7% increase in the weight percentage of carbides.

Figure 4c shows that none of the analysed factors has a significant effect on the percentage of precipitated K_2_ carbides. However, if a Pareto chart is used to represent the obtained effects, it can be seen that, although it does not show a significant effect, Factor F (tempering time) is the one that produces a greater effect on the percentage of these carbides (see Figure 5). Thus, placing this factor at its +1 level leads to a 7% by weight increase in carbide density. In this respect, the question may arise as to whether a high background of the diffractograms, due to the high fluorescence of the very Fe-rich compounds, might make it difficult to identify low intensity Bragg peaks belonging to carbides precipitated in a second destabilization of the austenite. Should this be the case, it might conceal the significant effect of Factor F on the percentage by weight of the precipitated carbides.

Figure 4d shows the significant effect of Factor A (destabilization temperature) on the volume of retained austenite: an increase in this temperature to 1100 °C leads to an increase in the said volume. This could be due to the increase in the solubility limit of C in the austenite.

Figure 4e shows, once again, that the destabilization temperature (Factor A) has a significant effect on the hardness of the material. Thus, there is an increase in the said hardness when this temperature is placed at its −1 level (1000 °C). This could be due to the increase in martensite (and the decrease in the amount of retained austenite) resulting from placing the destabilization temperature at 1000 ° C.

Figure 4f shows that Factor F (tempering time) has a significant effect on abrasive wear resistance: placing this factor at its −1 level (3 h) leads to an increase in the percentage wear. To increase the material’s resistance to erosive wear, the tempering time should be increased to 6 h (+1 level). This increase in wear resistance could be due to the second destabilization of austenite during tempering, resulting in the precipitation of secondary carbides, which would confirm the comments related to Figure 4c. This second destabilization requires a long time due to the difficulty of diffusion of the carbon atoms at the tempering temperatures.

**Figure 6:**
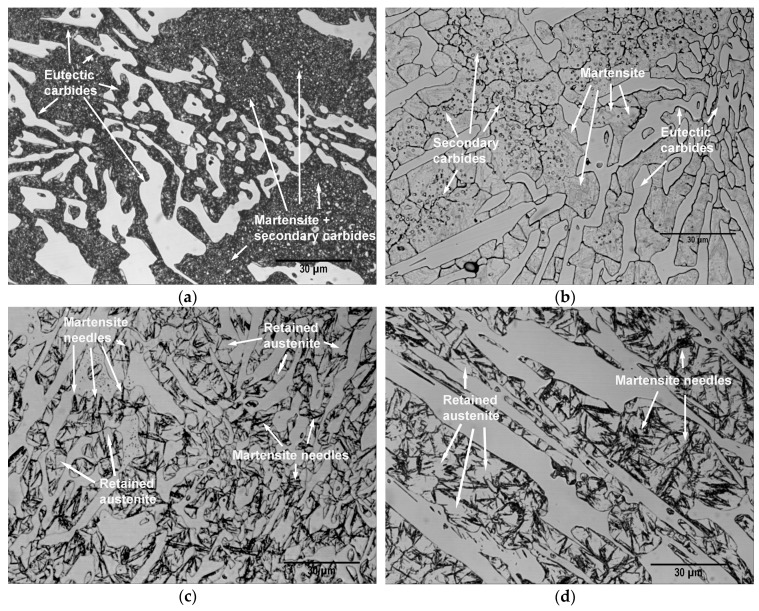
Representative micrographs of the microstructure obtained after the different heat treatments (all at ×1000 magnification): (**a**) Experiment 1; (**b**) Experiment 2; (**c**) Experiment 6; (**d**) Experiment 8.

Figure 6 shows the microstructure of some specimens obtained after the different heat treatments; in particular, those corresponding to Experiments 1, 2, 6 and 8. Figure 6a, which corresponds to Experiment 1, shows the majority presence of martensite. Figure 6b–d corresponding respectively to Experiments 2, 6 and 8, show a greater amount of retained austenite. Figure 6c,d, with a slightly longer exposure to the etching reagent than in Figure 6b, reveal the characteristic martensite needles embedded in the retained austenite. Secondary carbides which precipitated during the destabilization of austenite can be observed in Figure 6a,b.

**Figure 7:**
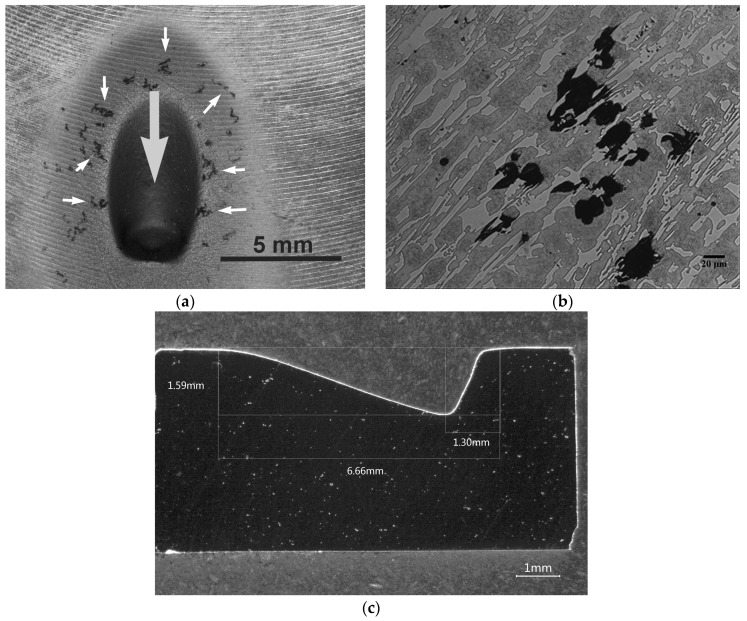
Morphology of the wear track. A track obtained from one of the specimens corresponding to Experiment 5: (**a**) The light grey arrow indicates the direction of impact of the corundum jet. The white arrows point to different impact marks of corundum particles around the wear track; (**b**) The impact of corundum particles produces deterioration of the matrix constituent without initially affecting the eutectic carbides. Once the deterioration of the matrix constituent surrounding these carbides occurs, they are released. (**c**) Profile of the wear track.

Figure 7 provides representative images of the morphology of one of the wear tracks corresponding to one of the specimens in Experiment 5. The white arrows in Figure 7a indicate impact marks of corundum particles, located in the vicinity of the main wear track. Figure 7b shows the microstructure in one of the regions adjacent to the wear track, where these impact marks appear. It can be seen that the impact of corundum particles initially produces deterioration of the matrix constituent (austenite and martensite) without affecting the eutectic carbides. Once the deterioration of the matrix constituent surrounding these carbides occurs, they are released. Figure 7c shows the profile of one of the wear tracks.

Bearing in mind this wear mechanism, it seems reasonable to conclude that the improvement in the erosive wear resistance of this white cast iron would be the result of an increase in the wear resistance of its matrix constituent. This improvement could be based on the development of new chemical compositions, together with changes in heat treatments, which allow the density of secondary carbides in the matrix constituent to be increased.

## 4. Conclusions

Based on the deliberate variation of parameters related to the heat treatment of a hypo-eutectic white cast iron containing 18% Cr and, in particular, taking into account those parameters related to the destabilization of austenite, it is concluded that:

1. The severity of the quench cooling medium does not significantly influence hardness, erosive wear resistance or the proportion of martensite or retained austenite.
2. The destabilization temperature is a key factor with respect to the percentage of retained austenite. In order to increase the amount of martensite and decrease the amount of retained austenite, low destabilization temperatures not exceeding 1000 °C are required. An increase of 100 °C in the destabilization temperature can lead to a 25% increase in retained austenite.
3. Moreover, tempering temperatures of around 500 °C favour an additional increase in the percentage of martensite.
4. Erosive wear commences on the matrix constituent without initially affecting the eutectic carbides. Once the deterioration of the matrix constituent surrounding these carbides occurs, they are released.
5. Long tempering times, of around 6 h, provide an increase in resistance to erosive wear due to a second destabilization of austenite during the said tempering. This destabilization delays the deterioration of the matrix constituent.

## Figures and Tables

**Table 1:** Composition (wt %).

C	Si	Mn	Cr	Mo
2.9	1.2	0.8	18.1	1.8

**Table 2:** Factors and Levels. The factors analysed in the Design of Experiments (DoE) are shown. These factors are denominated using the letters A to F.

Factors			Levels	
Code	Description of the Factors	Units	−1 Level	+1 Level
A	Destabilization temperature of austenite	°C	1000	1100
B	Dwell time at the destabilization temperature	h	4	8
C	Number of tempers	–	1	2
D	Quench cooling medium	–	air	oil
E	Tempering temperature	°C	200	500
F	Tempering time (h)	h	3	6

**Table 3:** Array of Experiments. The samples corresponding to each experiment were prepared by placing the analysis factors at the levels indicated in this array.

No.	A	B	C	D	E	F	Restricted Confounding Pattern
1	−1	−1	−1	+1	+1	+1	A + BD + CE B + AD + CF C + AE + BF D + AB + EF E + AC + DF F + BC + DE AF + BE + CD
2	+1	−1	−1	−1	−1	+1	
3	−1	+1	−1	−1	+1	-1	
4	+1	+1	−1	+1	−1	-1	
5	−1	−1	+1	+1	−1	-1	
6	+1	−1	+1	−1	+1	-1	
7	−1	+1	+1	−1	−1	+1	
8	+1	+1	+1	+1	+1	+1	

**Table:** (**a**) Austenite

No.	(111)		(200)		(222)	
	2θ (°)	I	2θ (°)	I	2θ (°)	I
1	43.657	2115	49.893	83	74.581	133
2	43.472	8041	50.395	737	74.438	367
3	44.110	4967	50.117	148	74.565	114
4	43.430	8417	50.183	575	74.562	121
5	43.529	5346	50.176	268	74.275	119
6	43.462	6454	50.276	532	74.171	95
7	43.466	6238	50.103	128	74.263	92
8	43.483	8096	49.469	116	74.368	217

**Table:** (**b**) Martensite

No.	(110)		(200)		(211)	
	2θ (°)	I	2θ (°)	I	2θ (°)	I
1	44.501	12203	64.743	270	82.118	1158
2	44.447	3817	65.095	183	82.632	319
3	44.498	6010	64.631	434	82.044	956
4	44.353	3029	64.733	180	82.142	224
5	44.431	6427	64.874	171	82.120	778
6	44.418	3521	67.232	108	82.167	383
7	44.438	5827	64.898	106	82.165	381
8	44.479	3675	64.926	104	82.374	321

**Table:** (**c**) M7C3 type Carbide

No.	2θ (°)	I	2θ (°)	I
1	39.417	1032	52.358	317
2	39.413	1035	52.391	399
3	39.354	814	52.356	148
4	39.351	378	52.292	186
5	39.354	731	52.219	175
6	39.337	591	52.358	317
7	39.405	798	52.319	163
8	39.413	803	52.405	221

**Table 5:** Microstructural parameters, weight distributions of the precipitated phases and volume of austenite.

No.	Rietveld Fitting	Phases	a (Å)	b (Å)	c (Å)	wt. %	Vol. (Å^3^)
1	R_wp_ = 9.54 R_exp_ = 6.67 Chi^2^ = 2.05	Martensite	2.87643	-	-	50.48 ± 1.59	-
		Austenite	3.59057	-	-	12.76 ± 1.17	46.290 ± 0.019
		K_2_ carbide	4.46111	6.99491	12.10891	36.76 ± 2.15	-
2	R_wp_ = 10.1 R_exp_ = 7.25 Chi^2^ = 1.95	Martensite	2.87511	-	-	10.05 ± 0.86	-
		Austenite	3.59862	-	-	42.2 ± 1.38	46.602 ± 0.004
		K_2_ carbide	4.46111	6.99491	12.10891	47.74 ± 2.25	-
3	R_wp_ = 10.0 R_exp_ = 6.72 Chi^2^ = 2.22	Martensite	2.87890	-	-	48.47 ± 1.61	-
		Austenite	3.58676	-	-	19.69 ± 1.39	46.143 ± 0.013
		K_2_ carbide	4.46111	6.99491	12.10891	31.83 ± 1.95	-
4	R_wp_ = 11.4 R_exp_ = 7.71 Chi^2^ = 2.18	Martensite	2.88817	-	-	9.34 ± 1.14	-
		Austenite	3.60308	-	-	61.09 ± 2.12	46.776 ± 0.005
		K_2_ carbide	4.46111	6.99491	12.10891	29.57 ± 2.03	-
5	R_wp_ = 10.5 R_exp_ = 7.37 Chi^2^ = 2.04	Martensite	2.88171	-	-	29.15 ± 1.32	-
		Austenite	3.59555	-	-	34.22 ± 1.53	46.483 ± 0.007
		K_2_ carbide	4.46111	6.99491	12.10891	36.63 ± 2.15	-
6	R_wp_ = 13.4 R_exp_ = 7.5 Chi^2^ = 3.2	Martensite	2.87831	-	-	10.52 ± 0.99	-
		Austenite	3.59951	-	-	49.58 ± 1.73	46.637 ± 0.005
		K_2_ carbide	4.46111	6.99491	12.10891	39.91 ± 2.16	-
7	R_wp_=11.6 R_exp_ = 7.05 Chi^2^ = 2.69	Martensite	2.88122	-	-	19.01 ± 0.96	-
		Austenite	3.58456	-	-	41.51 ± 1.36	46.445 ± 0.005
		K_2_ carbide	4.46111	6.99491	12.10891	39.48 ± 2.05	-
8	R_wp_ = 11.0 R_exp_ = 7.18 Chi^2^ = 2.35	Martensite	2.87366	-	-	1.09 ± 0.62	-
		Austenite	3.59837	-	-	56.95 ± 1.68	46.593 ± 0.004
		K_2_ carbide	4.46111	6.99491	12.10891	41.97 ± 2.22	-

**Table:** (**a**) Percentage by weight of the phases present

No.	Martensite		Austenite		M_7_C_3_		Calculated Effects
	(wt %)	Effect	(wt %)	Effect	(wt %)	Effect	
1	50.48	22.313	12.76	39.750	36.76	37.986	Average
2	10.05	−29.127	42.2	25.41	47.74	3.622	A+BD+CE
3	48.87	−5.472	19.69	10.12	31.83	−4.547	B+AD+CF
4	9.34	−14.742	61.09	11.63	29.57	3.022	C+AE+BF
5	29.15	0.402	34.22	3.01	36.63	−3.507	D+AB+EF
6	10.52	10.852	49.58	−10.01	39.91	−0.737	E+AC+DF
7	19.01	−4.312	41.51	−2.79	39.48	7.002	F+BC+DE
8	1.09	−0.047	56.95	2.97	41.97	3.112	AF+BE+CD

**Table:** (**b**) Volume of the austenite crystal cell

No.	Å^3^	Effect	Calculated Effects
1	46.29	46.496	Average
2	46.602	0.311	A+BD+CE
3	46.143	−0.013	B+AD+CF
4	46.776	0.086	C+AE+BF
5	46.483	0.078	D+AB+EF
6	46.637	−0.160	E+AC+DF
7	46.445	−0.027	F+BC+DE
8	46.593	−0.081	AF+BE+CD

**Table:** (**c)** Hardness and weight loss in the erosive wear test

No.	Hardness		Weight Loss		Calculated Effects
	HV100	Effect	mg	Effect	
1	833	781.75	79.23	83.095	Average
2	679	−142.5	80.95	−0.99	A+BD+CE
3	877	−1.5	86.13	0.52	B+AD+CF
4	693	22.5	83.25	1.41	C+AE+BF
5	859	−15.5	86.73	−0.7	D+AB+EF
6	759	26.5	84.43	−0.41	E+AC+DF
7	843	−30.5	82.27	−4.08	F+BC+DE
8	711	−0.5	81.77	1.6	AF+BE+CD
